# Best Practices for Non‐Pharmacological Treatments for Older Adults in the Geriatric Mental Health Outpatient Services (GMHOS) at the Centre for Addiction and Mental Health (CAMH)

**DOI:** 10.1002/alz70858_101283

**Published:** 2025-12-25

**Authors:** Senaya Karunarathne, Kimberly Martin, Dewi Clark, Ann‐Sylvia Brooker, Mackenzie Hilton, Jordanne Holland

**Affiliations:** ^1^ Centre for Addiction and Mental Health, Toronto, ON, Canada

## Abstract

**Background:**

The Geriatric Mental Health Outpatient Services (GMHOS) at the Centre for Addiction and Mental Health (CAMH) in Toronto, Ontario, deliver evidence‐based care to older adults with complex mental health conditions. GMHOS currently offers limited group programming tailored to the unique needs of its four outpatient clinics: Neuropsychiatry, Memory, Late Life Anxiety and Mood Disorders and General Geriatrics. Existing therapies, such as “Learning the Ropes” and “Goal Management Training,” address cognitive and emotional needs but leave significant gaps for conditions like anxiety, depression, and dementia. Challenges related to resources and accessibility further constrain scalability.

This quality improvement project aims to (1) gather insights from current GMHOS program facilitators regarding usability, satisfaction, effectiveness, and areas for improvement, and (2) conduct a literature review of academic research to identify evidence‐based, nonpharmacological interventions delivered in outpatient settings that can enhance mental health outcomes for geriatric patients.

**Method:**

A mixed‐methods approach was employed. Structured questionnaires were administered to GMHOS group facilitators to collect feedback on current programming using validated recovery‐oriented tools, including the American Association of Community Psychiatrist Recovery‐Oriented Service Evaluation (AACP‐ROSE) and the Consumer Recovery Outcomes System (CROS). A comprehensive literature search was also conducted by a research librarian using PubMed and PsycINFO databases to identify evidence‐based nonpharmacological therapies for outpatient geriatric mental health care.

**Result:**

Facilitators identified strengths in current programs, such as reminiscence therapy and art‐based interventions, in promoting engagement and emotional well‐being. However, they identified gaps in addressing cultural diversity, scalability, and accessibility. Suggested improvements included participant‐driven activities, structured goal‐setting, and enhanced staff training. Preliminary findings from the literature review identified therapies such as Yoga‐Cognitive Behavioral Therapy (Y‐CBT), Cognitive Stimulation Therapy (CST), mindfulness‐based interventions, and life review therapy as effective for addressing conditions like anxiety, depression, and dementia.

**Conclusion:**

Facilitator feedback and evidence from the literature emphasize the importance of tailoring interventions to participant needs while fostering scalable and inclusive approaches. Integrating evidence‐based therapies like Y‐CBT and CST alongside enhancements to existing programs can address critical service gaps in GMHOS. Future steps include piloting new therapies, evaluating their long‐term impacts, and ensuring sustained improvement in outcomes for geriatric outpatients.

